# Effect of the Nucleophile’s Nature on Chloroacetanilide Herbicides Cleavage Reaction Mechanism. A DFT Study

**DOI:** 10.3390/ijms22136876

**Published:** 2021-06-26

**Authors:** Sebastián A. Cuesta, F. Javier Torres, Luis Rincón, José Luis Paz, Edgar A. Márquez, José R. Mora

**Affiliations:** 1 Grupo de Química Computacional y Teórica (QCT-USFQ), Departamento de Ingeniería Química, Universidad San Francisco de Quito, Diego de Robles y Vía Interoceánica, Quito 170901, Ecuador; sebascuestahoyos89@gmail.com (S.A.C.); jtorres@usfq.edu.ec (F.J.T.); lrincon@usfq.edu.ec (L.R.); 2 Departamento de Ingeniería Química, Instituto de Simulación Computacional (ISC-USFQ), Universidad San Francisco de Quito, Diego de Robles y Vía Interoceánica, Quito 170901, Ecuador; 3 Departamento Académico de Química Inorgánica, Facultad de Química e Ingeniería Química, Universidad Nacional Mayor de San Marcos, Cercado de Lima 15081, Peru; jpazr@unmsm.edu.pe; 4 Grupo de Investigaciones en Química y Biología, Departamento de Química y Biología, Facultad de Ciencias Exactas, Universidad del Norte, Carrera 51B, Km 5, Vía Puerto Colombia, Barranquilla 081007, Colombia

**Keywords:** chloroacetanilide herbicides, nucleophilic substitution, reaction force, electronic flux, DFT calculations

## Abstract

In this study, the degradation mechanism of chloroacetanilide herbicides in the presence of four different nucleophiles, namely: Br^−^, I^−^, HS^−^, and S_2_O_3_^−2^, was theoretically evaluated using the dispersion-corrected hybrid functional wB97XD and the DGDZVP as a basis set. The comparison of computed activation energies with experimental data shows an excellent correlation (R^2^ = 0.98 for alachlor and 0.97 for propachlor). The results suggest that the best nucleophiles are those where a sulfur atom performs the nucleophilic attack, whereas the other species are less reactive. Furthermore, it was observed that the different R groups of chloroacetanilide herbicides have a negligible effect on the activation energy of the process. Further insights into the mechanism show that geometrical changes and electronic rearrangements contribute 60% and 40% of the activation energy, respectively. A deeper analysis of the reaction coordinate was conducted, employing the evolution chemical potential, hardness, and electrophilicity index, as well as the electronic flux. The charge analysis shows that the electron density of chlorine increases as the nucleophilic attack occurs. Finally, NBO analysis indicates that the nucleophilic substitution in chloroacetanilides is an asynchronous process with a late transition state for all models except for the case of the iodide attack, which occurs through an early transition state in the reaction.

## 1. Introduction

The presence of weeds and grasses competing with plants for resources such as nutrients, water and sunlight has become a serious agricultural problem that hampers high yields in crops [1]. Reductions in the production yields as high as 80% resulted in big concerns regarding the growth control of these undesirable plants from the farm fields [2]. The use of chemical herbicides is considered one of the most efficient methods to prevent weed invasion, being an essential and common practice in modern agriculture [1,2,3].

Chloroacetanilides, first synthesized in the 1960s, represent a family of herbicides that includes products such as: metolachlor, acetochlor, butachlor, and alachlor. Additionally, this family of compounds represents one of the most widely used pesticides worldwide to fight small-seeded broadleaf weeds and annual grasses [4,5]. The use of these compounds, and accordingly their production, has significantly increased since 1980. Moreover, they are used in the production of several economically important crops such as corn, sugar cane, cotton, beetroot, maize, rice, sunflower, and soybean crops [6,7]. The particular case of acetochlor, metolachlor, and alachlor reach the 4th (~33 million lb), 10th (~18 million lb), and 16th (~8 million lb) place in the most widely used agricultural chemicals in the US, according to the United States Environmental Protection Agency (US EPA) [7,8]. In addition, their use has been observed to reach countries in Africa, Europe, Latin America [3,9]. China is also a great consumer of chloroacetanilide herbicides, using around 10,000 metric tons every year [4].

It has been suggested that weeds have become more resistant to these substances. This has resulted in the need for larger amounts of pesticides to achieve the same weed control. The latter represents a risk not only to the agricultural land but to the environment itself, where an accumulation have been determined in continental and marine natural waters [1,10]. Chloroacetanilide and its metabolites have been found in ground and surface water due to their relatively high water solubility [4,10]. Acetochlor has been detected in shallow groundwater up to one year after its application, being the third most frequently detected herbicide behind glyphosate and atrazine [5,10,11]. Due to its transport mechanism, which includes surface runoff, soil erosion and leaching, scientists estimate that around 95% of freshwater streams near farming fields contain detectable quantities of acetochlor [8,12]; thus, strict regulations have been imposed for acetochlor, whose concentration in water cannot exceed 2 ug/L. Maximum concentration values for other chloroacetanilides have not been established; however, other members of the chloroacetanilides family, as metolachlor, are already present in the EPA’s “Drinking Water Contaminant Candidate List” [11]. Given the latter, the removal of these type of herbicides from wastewater and aquatic ecosystems has become imperative [4].

In addition to the environmental issues, some chloroacetanilide herbicides have been identified as probable human carcinogens. In this sense, alachlor and acetochlor have been classified as B2 class substances (i.e., likely human carcinogens) by the US EPA, while metolachlor is classified as a C class substance (possible human carcinogen) [13,14]. Their accidental consumption has been associated with eye, liver, kidney, spleen problems and anemia [7]. Indeed, recent studies have shown significant rates of the carcinogenic nature of acetochlor in rats, tadpoles and minnows causing stomach, liver, bone, thyroid and lung tumors [3]. Furthermore, these substances exhibit moderate to high chronic toxicity to aquatic vertebrates and invertebrates; and high toxicity to aquatic plants and some green algae [8,15]. Currently, the carcinogenic mechanism of chloroacetanilide compounds has not been elucidated; however, in vitro ecotoxicological evidence suggests that the carcinogenic properties are related to the ability of the herbicide to undergo a nucleophilic reaction, causing changes and damages in the DNA [13]. This has increased the concern of being able to identify and determine the effect of these compounds and their metabolites in the environment [3,8]. 

It is important to point out that chloroacetanilides are recalcitrant substances; thus, their degradation occurs very slowly in soil–water systems, while in aquifer environments, is almost negligible [5,12,13]. The oxanilic acid and ethane sulfonic acid (ESA) derivatives of alachlor, acetochlor and metolachlor are usually detected in higher concentrations, being identified as the major byproduct of chloroacetanilide herbicides in water bodies [4,16,17]. Still, their transformation rate is estimated to be very slow under natural conditions. Recent studies have reported that some sulfur compounds could rapidly react with chloroacetanilide herbicides, turning them into less toxic products [18,19], and this mechanism has been suggested as an effective approach for chloroacetanilide remediation in soil and aqueous environments. In these regards, the degradation mechanism of chloroacetanilide compounds can be simply foreseen as the substitution of the chlorine atom by a different functional group through an intermolecular S_N_2 process [9,14]. In order to support this statement, a computational study of chloroacetanilide herbicides degradation mechanism using HS^−^ ion as a nucleophile, by means of electronic structure calculations performed at the wB97XD/6-311++G(2d,2p) level, was presented in a previous study in order to obtain insights into the degradation process. Three different mechanisms were evaluated being the bimolecular nucleophilic substitution (S_N_2) mechanism, with the most favorable one having activation free energies around 20 kcal/mol [13]. Under this approach, other anionic nucleophiles present in water bodies such as iodide and bromide ions have been experimentally considered for the S_N_2 nucleophilic substitution reaction of chloroacetanilide [20]. 

**Scheme 1:**
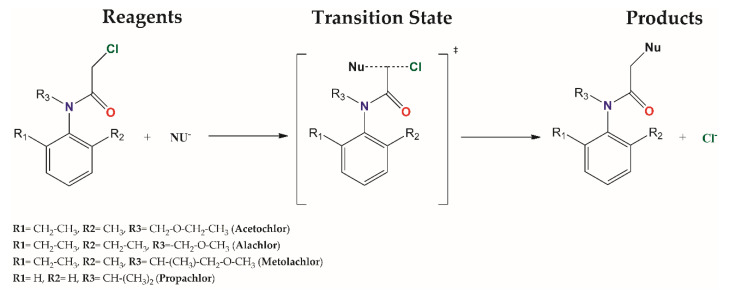
S_N_2 reaction mechanism of chloroacetanilides.

In this study, the effect of the nucleophile nature in the S_N_2 mechanism, which has been reported as the most favorable mechanism for these reactions (Scheme 1), was theoretically evaluated in detail by employing the Density Functional Theory (DFT) with the long-range dispersion-corrected Head–Gordon hybrid functional ωB97XD. Four well-known chloroacetanilide herbicides were studied using four different nucleophiles, taking into account the geometrical and electronic rearrangement occurring on the reaction pathway.

## 2. Results and Discussion

**Figure 1:**
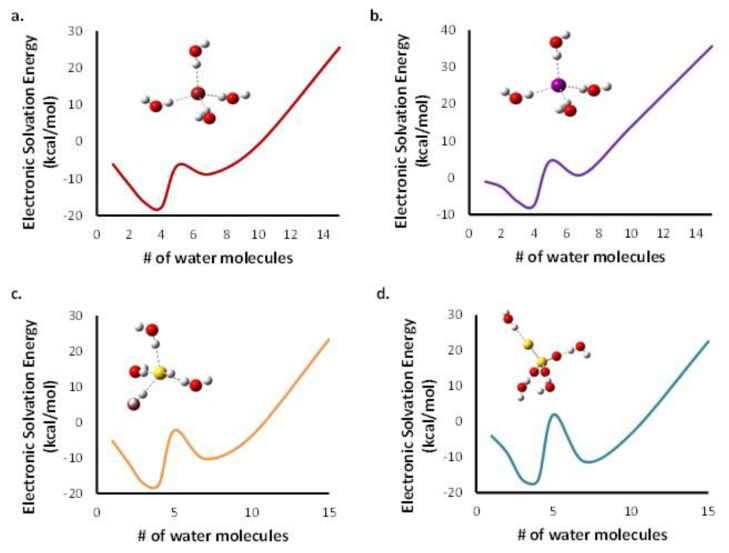
Solvation electronic energy of bromide (**a**), iodide (**b**), bisulfide (**c**), and thiosulfate (**d**) ions using a different number of water molecules.

Following Scheme 1, the reaction of each chloroacetanilide with the four different nucleophiles was studied and their activation free energies calculated. It is known that nucleophiles act differently in protic and aprotic solvents. In protic solvents, I^−^ ion is a better nucleophile than Br^−^, while the opposite is expected in aprotic solvents [21]. The results obtained using the solvation model density (SMD) did not agree with the aforementioned because Br^−^ presented a lower activation barrier (~10 kcal/mol) than I^−^ in all studied models. Probably, this failure is due to the absence of local interactions with the protic solvent molecules within the SMD approach. To circumvent this problem, the effect of including water molecules to simulate an explicit solvent around the reactant, nucleophile, and transition state (Figure S1) is evaluated. In the chloroacetanilides and transition states, the effect of an explicit solvent is very small (Table S1) because they are not charged species cancelling out when estimating the activation free energy. In order to reproduce the solvation effect, systems with different numbers of water molecules around the nucleophile were built and tested. Regarding the latter, the model with four water molecules generated the most stable complexes, forming a tetrahedral environment around the anion (Figure 1). From five molecules onwards, water molecules showed a preference to form internal hydrogen bonds rather than interact with the ion. All four nucleophiles presented an analogous energy profile, where the electronic solvation energy of −17.7 kcal/mol was estimated for Br^−^, −7.51 for I^−^, −17.6 kcal/mol for HS^−^, and −16.54 kcal/mol for S_2_O_3_^−2^.

Considering the solvation free energy value of the different models employing a four water molecule system, the activation free energies were estimated (Table 1). For the thiosulfate nucleophile (S_2_O_3_^−2^), two options were taken into account: one where the nucleophilic attack occurs through the sulfur atom, and the other through the oxygen atom.

**Figure 2:**
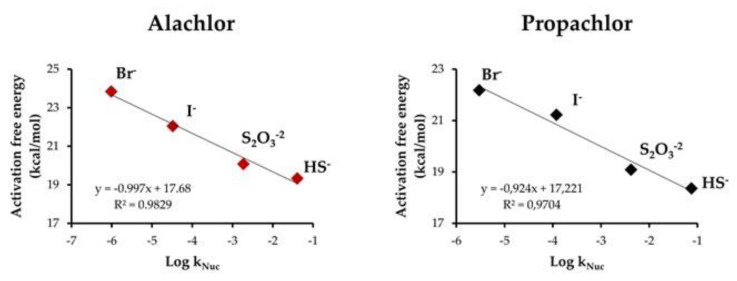
Log knuc vs. activation free energy for alachlor and propachlor. R^2^ = coefficient of determination, y = activation free energy, x = log k_nuc_.

Regarding acetochlor, the most favorable S_N_2 reaction with thiosulfate occurs through the sulfur atom instead of the oxygen one having a difference in activation free energy of around 7 kcal/mol. This was expected, as in protic solvents, sulfur is always a better nucleophile than oxygen. On the other hand, comparing the two halogens studied, the iodide anion presents lower activation free energies, acting as a better nucleophile than bromide. The bisulfide anion was observed to be slightly less reactive than the thiosulfate ion. The opposite was observed for the other chloroacetanilides, where this anion was observed to produce the lowest activation energies with values close to ~20 kcal/mol, and it was in good agreement with experimental reports [14]. In this regard, the calculated activation free energy for alachlor and propachlor were compared with the logarithm of the experimental rate constant (Knuc) values obtained by Lippa et al. [14]. As a result, a linear relationship was found with a coefficient of determination (r^2^) of 0.98 for alachlor and 0.97 for propachlor (Figure 2) which means that the model proposed in this article adequately describes these particular reactions. The obtained linear equations with a slope near to 1 and an intercept of about 17–18 suggest that both systems are degraded by the same mechanism, with the HS^−^ being the best nucleophile with a high k_nuc_. The intercept values for both systems are similar and are related to the entropy changes occurring during the formation of the transition state, which imply a bimolecular reaction with a great loss in translational degrees of freedom. It is important to indicate that, for the case of acetochlor and metolachlor, experimental results for the attack of all nucleophiles considered in this work are not reported.

**Figure 3:**
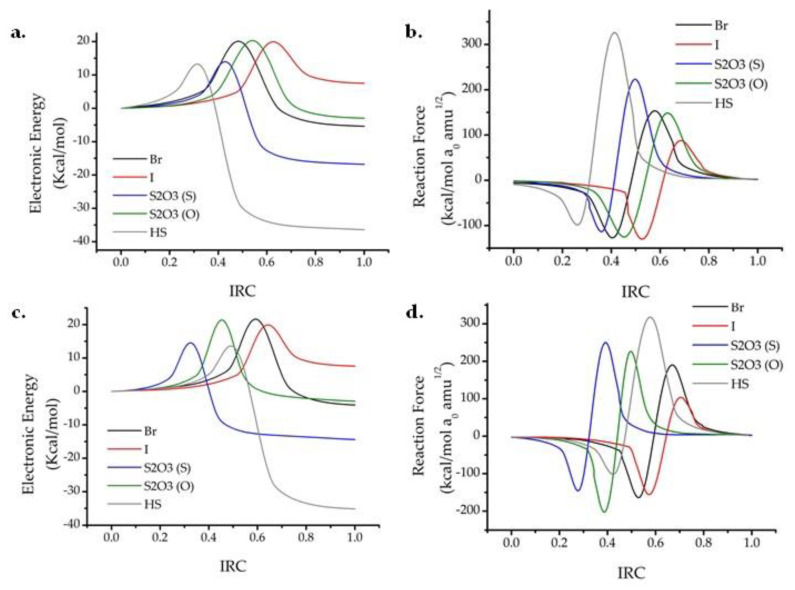
IRC and RF plots for the nucleophilic substitution of alachlor (**a**,**b**) and propachlor (**c**,**d**).

Each one of the previous reactions was further analyzed through an intrinsic reaction coordinate (IRC) calculation, the reaction force (RF), as well as the reaction electronic flux (REF) descriptors. The IRC plots show the energy profile of the nucleophilic substitution mechanism from the reagents to the products. From the IRC graph, the RF plot is obtained (Figure 3). Due to the similarity of the models, only alachlor and propachlor plots are shown. The figures for the other two models are presented in the Supporting Information (Figures S2 and S3). 

**Figure 4:**
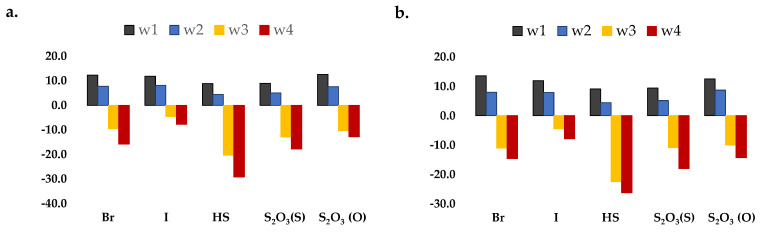
Reaction force works for the nucleophilic substitution reaction of alachlor (**a**) and propachlor (**b**).

Looking at the electronic energy profile, only the I^−^ model produce products that are less stable than its reagents. The HS^−^ ion produces the most stable product and presents the lowest activation energy, being the best nucleophilic agent followed by the S_2_O_3_^−2^ anion. The RF plots obtained agrees with a classical concerted mechanism, where four reaction works, two describing geometrical rearrangements and two describing electronic changes (see the definition in the Section 3), were estimated. The reaction works for all the models were calculated and tabulated in Tables S2–S5. Values for the different works in alachlor and propachlor are depicted in Figure 4. The graphs for the other models are available in the Supporting Information (Figures S4 and S5).

The results show that for all models, the geometrical changes contribute around 60% (associated to w1, black bar in Figure 1) and the electronic changes 40% (associated to w2, blue bar in Figure 1). The main geometrical changes occur on the carbon atom where the two hydrogens align to the plane in order to let the chlorine atom leaves and the nucleophiles enter the system in a forward mechanism [22]. The distance between the nucleophile and the carbon where the attack is being performed was found to be the same at the transition state (TS) structure independently of the chloroacetanilide studied. This distance is directly influenced by the atomic radius of the element performing the attack. In this sense, an r^2^ value of 0.95 was found when correlating the distance at the transition state with the atomic radius of the element. Electronic changes occur when the nucleophile enters in the system and is close enough (i.e., C-Cl distance of ~2.0 Å and C-Nu distance of ~2.75 Å) to produce charge rearrangements. Here, the C-Cl bond started weakening, resulting in its cleavage, while the C-Nu interaction became stronger, forming a bond. The smallest values of w1 and w2 were obtained for the case of the HS^−^ as nucleophile, in consonance with the smallest activation energies found for those systems.

**Figure 5:**
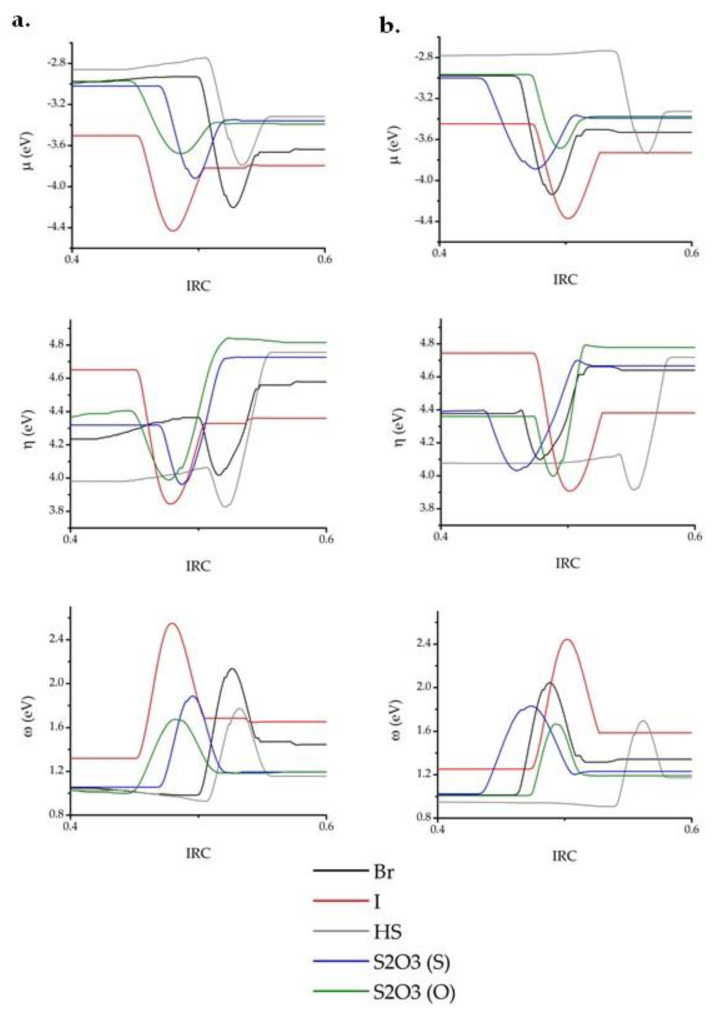
µ, η, and ω zoomed plots for the nucleophilic substitution of alachlor (**a**) and propachlor (**b**).

In order to obtain further insights in the changes described above and evaluate the reactivity of the system and some electronic events occurring as the reaction proceeds, three molecular descriptors, chemical potential (µ), hardness (η), and electrophilicity (ω), were plotted along the reaction coordinate. Figure 5 shows the plots for the alachlor and propachlor systems at the transition state region (see the definition in the Section 3), while the complete plots can be found in the Supporting Information (Figure S6), along with the graphs for the other systems (Figures S7 and S8).

It is observed that the chemical potential and the hardness are linearly related, while electrophilicity is inversely related. Furthermore, energy (E) is also inversely related to µ and η. In this sense, E and ω reach a maximum at the TS, while µ and η reach a minimum. As the chemical reaction proceeds, µ, η, and ω remain stable during the reagent’s region, confirming, that in this region, geometrical rearrangements are mainly taking place. During the TS region, where all the electronic changes occur, there is a small increase in µ and η, followed by a rapid decrease up to the TS structure. In ω, the behavior is the opposite. From a thermodynamic point of view, this decrease in µ infers the chemical process is spontaneous [23]. Regarding η, the decrease was expected because, during the TS, the system is in a meta-stable state where the HOMO-LUMO gap is the shortest, fulfilling the Maximum Hardness Principle [24]. Once in the products region, the three descriptors stabilize again, and a geometrical relaxation process occurs. 

The TS structure has the lowest η value, indicating is the most unstable structure [23]. Each descriptor affects the mechanism in a different level and to a different extend. That is why E and µ values at the TS structure are not correlated at all. In this sense, although the HS^−^ model has the lowest activation energy, its µ at the transition state structure is not highest, nor the lowest. It has been reported that the activation process is governed by changes in µ, while the relaxation process is governed by by changes in η [25]. The TS can be considered the structure with maximum energy and electrophilicity, and minimum chemical potential and hardness.

**Figure 6:**
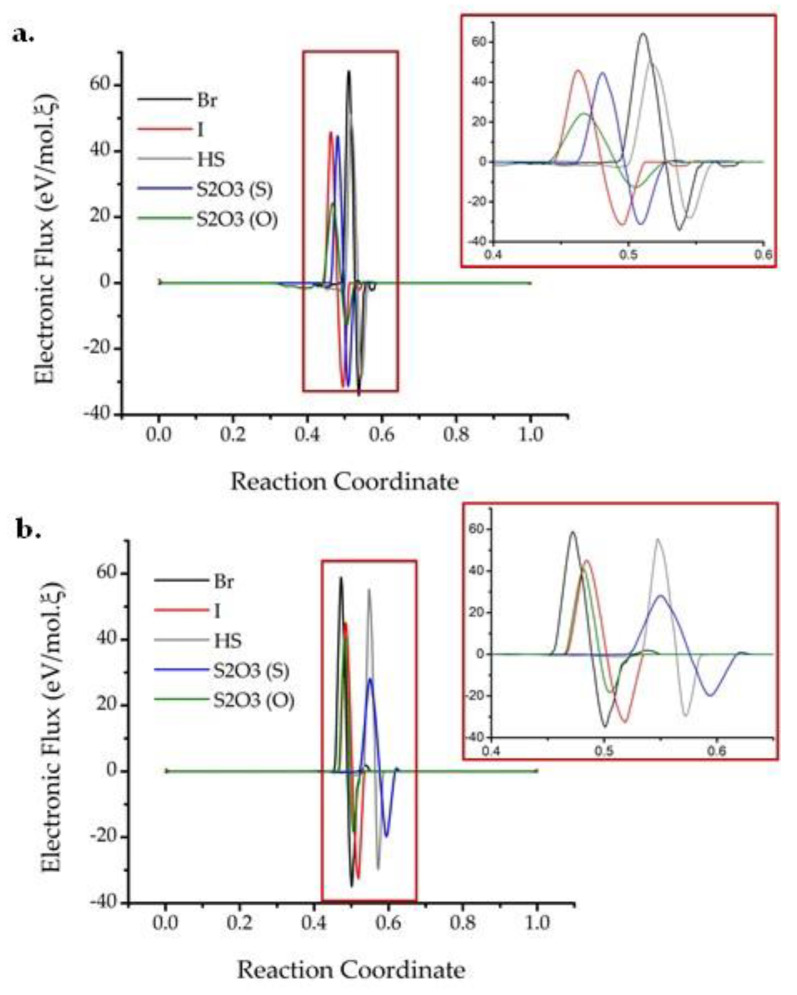
REF plots for the nucleophilic substitution of alachlor (**a**) and propachlor (**b**).

REF profiles were plotted to obtain insights on the electronic rearrangement occurring along the reaction. Alachlor and propachlor systems are shown in Figure 6, while acetochlor and metolachlor are shown in Figures S9 and S10, respectively.

By analyzing the REF for the different models with the different nucleophiles, it is clearly observed that the shape of all the profiles is similar, meaning an analogous electronic flux is followed by all the studied systems. A similar profile is reported in the literature [26] suggesting that this type of profile is typically observed in S_N_2 reactions. The differences found are attributed to the bond strengths caused by the atom’s nature. In the reactant region (R–ξ1), no electronic flux was found. These results correlate with the RF profile, where this portion is attributed to geometrical contributions, suggesting only electrostatic interactions occur between the nucleophile and the carbon atom. When the reaction enters into the TS region (ξ1–ξ2), where all the electronic redistribution takes place according to the RF profile, an important increase in electronic flux can be seen. This event activates the reaction which prepares the system for both the C-Nu bond formation and the C-Cl bond cleavage. No electronic flux was observed at the transition state structure due to a cancellation carried out by the equilibration between the nucleophile and the leaving group. This occurs despite it being a thermodynamically unstable structure. In the subsequent region, electronic flux is again observed until the reaction reaches the products region (ξ2–P) where the flux disappears, letting electrostatic and geometrical changes relax the system up to the final product. In a REF profile, positive values are associated with spontaneous changes in the electronic density as in a bond strengthening process. For non-spontaneous electronic processes, such as the ones that occur during bong weakening, the electronic flux reflects negative values. In this sense, as the reaction proceeds, it is observed that there is a spontaneous electronic change caused by the approach of the nucleophile towards the carbon atom that causes the C-Cl bond to start weakening until its cleavage. This process involves most of the electronic rearrangements; accordingly, it presents the highest REF value in the graph. After this point, the flux tends to decrease up to the TS, where it becomes a non-spontaneous electronic process up to a minimum where the nucleophile enters the system, and the chlorine atom leaves. Although there still is a flux, it is lower in every case from the one occurring at the beginning of the TS region. After this minimum point, a spontaneous process that includes the C-Nu bond strengthening occurs up to a stable electronic conformation, where no more flux is observed.

**Figure 7:**
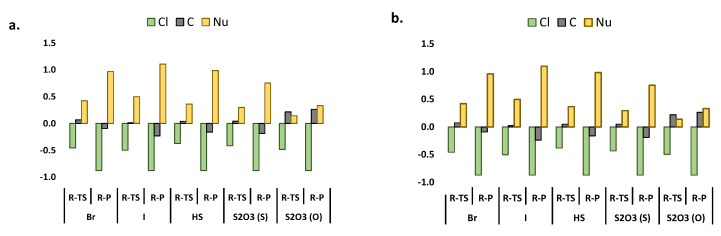
Charge change from reagent to the transition state (R-TS), and from the reagent to the product (R-P) for the nucleophilic attack of alachlor (**a**) and propachlor (**b**).

A complementary charge analysis was performed for all models and tabulated in Tables S6–S9. As the behavior in all models is very similar between the different models, only alachlor and propachlor are plotted in Figure 7. As the nucleophilic substitution of chloroacetanilides proceeds from the reagents to products, the chlorine atoms gain electron density. The different R groups in chloroacetanilides have a negligible influence on the mechanism itself; therefore, it was found that for all studied models, chlorine electron density increases −0.88. On the other hand, the nucleophiles lose electron density, with iodide being the one that loses the most (~1.11) followed by bisulfide (~0.99) bromide (~0.97), thiosulfate attack through the sulfur atom (~0.75) and the oxygen atom (~0.33). In the case of the carbon atom, it increases the electron density in all models except for the oxygen attack of the thiosulfate.

Finally, Wiberg bond indexes (Tables S10–S13) were estimated to obtain synchronicity and evolution percentage values. From the results, the C-Cl bond dissociation was found to be the most important event to reach the TS with higher evolution than the C-NU bond in all studied models. The synchronicity and average evolution percentage values for alachlor and propachlor are shown in Table 2. The highest evolution for the C-Cl bond was found for the iodide models which can be reflected in its synchronicity value (~0.92) and the %Evav (~56.5%), which are the highest too. The evolution percentage for the C-Cl compared to C-Nu bond shows a difference between 8% and 10% for all models except S_2_O_3_^−2^ (O) which presents a 15% difference. For the studied models, only iodide presents a late transition state (%Evav < 50%) while the others present an early TS, with HS^−^ being the earliest (41.8%). All models are shown to be slightly asynchronous, with values between 0.839 and 0.928.

## 3. Materials and Methods

**Figure 8:**
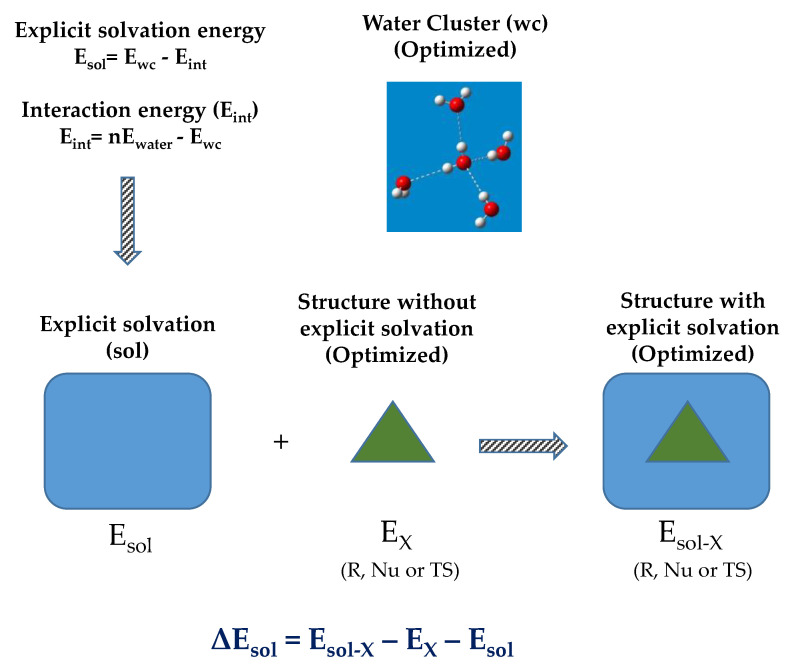
Schematic representation of the solvation model approach used in this work.

Four chloroacetanilide compounds were evaluated according to Scheme 1. To perform the nucleophilic substitution process, four anions were evaluated (bromide, iodide, bisulfide and thiosulfate). All the calculations were performed using the DFT long-range dispersion-corrected Head–Gordon hybrid functional ωB97XD [13,27,28,29], as implemented in Gaussian 16 [30]. The DGDZVP basis set was used to express the wavefunction, since it has been determined to correctly describe the H-Xe elements range [31,32,33,34,35]. Solvent effects were also considered by employing both implicit method and explicit water molecules. For the implicit method, a Polarizable Continuum Model (PCM) of water was used within the solvation model density (SMD) approach [36,37]. On the other hand, as nucleophiles behave differently in protic and aprotic solvents [21], solvation electronic energies were obtained using water molecules in an explicit way to determine the most stable system around the reactants. Figure 8 presents a schematic representation of this approach.

For all the systems studied, the reagents (R), transition state (TS) and products (P) structures were optimized. Frequency calculations were performed to characterize the stationary points. The Berny algorithm was employed to find the correct TS where the only imaginary frequency found had to be linked to the nucleophilic attack and chlorine atom removal event [38]. The activation free energy was estimated by subtracting the Gibbs free energy obtained for the transition state structure minus the Gibbs free energy of the reactants. A molecularity correction of −1.89 kcal/mol was added to the activation free energy, because it is a bimolecular system [39]. Computational results were compared against experimental kinetic results obtained by Lippa et al. using pseudo-first-order conditions with a nucleophile concentration ranging from 1.3 nM to 1M, and analyzed using gas chromatograpgy [14]. 

Intrinsic reaction coordinate (IRC) calculations were conducted around all the TS structures in order to obtain all the structures that connect the reagents with the products [40,41,42]. Then, the reaction force quantum mechanical descriptor was used to further characterize the reaction. The reaction force (RF) profile of each reaction was obtained by taking the derivative of the energy profile, E(ξ), over the normalized reaction coordinate (Equation (1)) [13,25,43,44].

(1) $$F\left(\xi \right)=-\frac{\mathrm{dE}}{d\xi }$$

**Figure 9:**
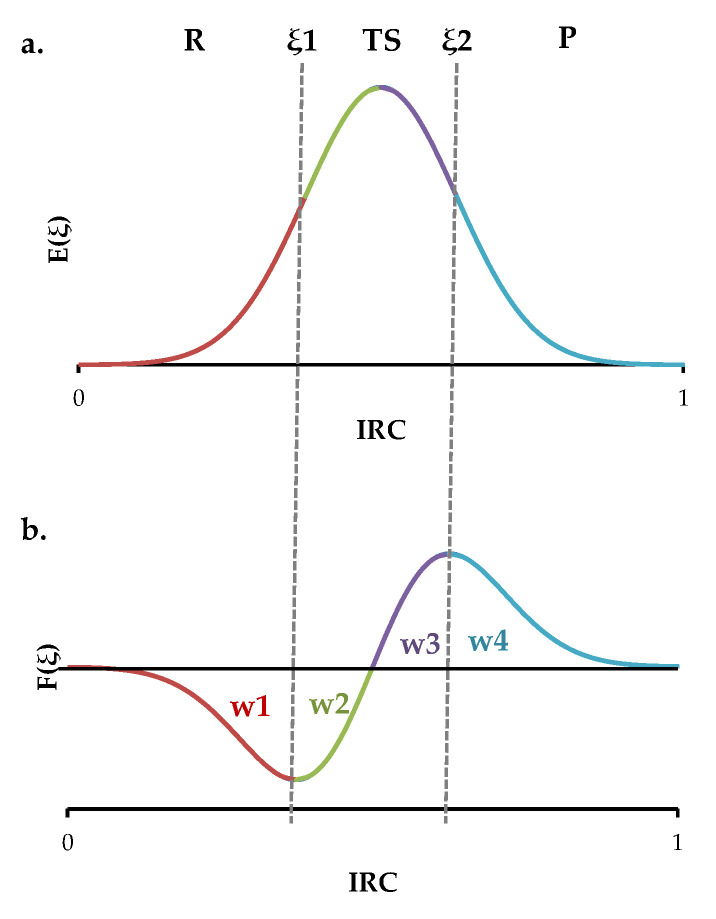
Potential energy profile (**a**) and reaction force profile (**b**) for a single step reaction along the IRC.

In a typical one step S_N_2 reaction, the RF plot has three regions separated by two critical points (ξ1 and ξ2) with the TS between both (dotted lines in Figure 9). The first one is called the reagents region, where geometrical rearrangements take place in order to go from the reagent to an activated state of them. The second zone, called the transition state region, starts at an activated state of the reagents, goes through the transition state, and ends at an activated state of the products. This region starts at a minimum (ξ1) in the RF plot and ends in a maximum (ξ2). Finally, the last section is called the products region, which describes the relaxation process going from the activated products to the final basal state of them [43,45,46,47]. By integrating each part of the profile, four different reaction works (Equation (4)) associated with the geometric and electronic contribution can be estimated, gaining an insight on the mechanism (Figure 9b). The first work (w1) is associated to geometrical changes, and the second work (w2) to the electronic ones occurring to the reagents in order to reach the TS. On the other hand, Work 3 (w3) estimates the electronic contribution required in the relaxation process, while Work 4 (w4) the geometrical ones. Negative signs in w3 and w4 means the system is relaxing [48,49,50].

(2) $$W_{x}=-\int_{i}^{j}F\left(\xi \right)d\xi .$$

Three additional molecular descriptors, i.e., chemical potential (µ), hardness (η), and electrophilicity (ω) described elsewhere [51], were used to obtain a deep understanding of the electronic events and chemical reactivity occurring during the reaction mechanism. Although the chemical potential (μ) is defined as the derivative of the electronic energy against the number of electrons (N) [43,52], it can also be defined in terms of the energy of the frontier Molecular Orbitals LUMO (Lowest Unoccupied Molecular Orbital) and HOMO (Highest Occupied Molecular Orbital), applying Koopmans’ theorem and the finite difference approximation described elsewhere [53,54]. The same can be carried out with η ($\frac{{Ɛ}_{\mathrm{LUMO}}-{Ɛ}_{\mathrm{HOMO}}}{2}$), defined as the first partial derivative of µ with respect to N, where a hardness profile can be built [55]. Hardness is a good descriptor when describing reaction mechanisms following the Maximum Hardness Principle, stating that at equilibrium, chemical species are as hard as possible [24]. In most cases, there is a relation between hardness and activation energy, where higher η means lower activation energies and also lower polarizability [56]. Furthermore, it allows one to study the solvent effect [57]. Finally, electrophilicity is a widely used reactivity parameter defined by Parr et al. [58], being successful in describing different organic systems, including Friedel–Craft, Diels–Alder, and 1,3 cycloaddition reactions [59,60,61]. A polarizable environment influences electrophilicity, where solvation enhances neutral compounds’ electrophilicity while attenuating charged ones [62].

Furthermore, the reaction electronic flux (REF) profile was constructed in order to gain a better understanding of the electronic changes occurring during the S_N_2 reaction. The REF was built by taking the derivative of μ over the normalized IRC (Equation (3)) [63,64].

J(ξ) = −dμ/dξ(3)

A natural bond orbital (NBO) analysis was also carried out on every system in order to evaluate the bond order evolution during the nucleophilic attack as the reaction proceeds [65,66]. Wiberg bond indexes (Bi) [67,68,69,70] and the evolution percentage (%Evi) of the bonds for the three atoms directly involved in the nucleophilic substitution were estimated (Equation (4)). With this information, the average bond evolution percent (%EVav) can be calculated, providing insights on how early or late in the reaction the transition state occurs in the reaction.

(4) $${\%\mathrm{Ev}}_{i}=\left[\frac{B_{i}^{\mathrm{TS}}-B_{i}^{R}}{B_{i}^{P}-B_{i}^{R}}\right]\times 100$$

Equation (5) allows the calculation of the synchronicity (Sy) of the system. This concept proposed by Moyano et al. describes if a process is synchronous (values around 1) or asynchronous (values around 0) [49,71,72,73].

(5) $$\mathrm{Sy}=1-\frac{\left[\sum_{i=1}^{n}\frac{\left|{\%\mathrm{Ev}}_{i}\;-{\;\%\mathrm{Ev}}_{\mathrm{av}}\right|}{{\%\mathrm{Ev}}_{\mathrm{av}}}\right]}{2n-2}$$

## 4. Conclusions

The S_N_2 mechanism of four different nucleophiles studied at the wB97XD level of theory and a DGDZVP basis set shows that sulfur atom attack is the most favorable mechanism, followed by iodine, bromine, and oxygen atoms. Activation free energies computed for all models are between 18 and 26 kcal/mol. A good correlation with experimental data implies the confidence of this methodology in the study of this type of reactions. To reach the transition state, all models show a 60% contribution of geometrical changes and 40% contribution of the electronic ones. The analysis of the reaction coordinate performed using the evolution of µ, η, and ω throughout the reaction shows the Maximum Hardness Principle is achieved, with the TS structure having the lowest η value. η and µ are linearly related, while ω and energy are inversely related. The most important electronic rearrangement occurs when the nucleophile approaches the system in a spontaneous process while the second one occurs during the carbon–nucleophile bond formation and carbon–chlorine bond cleavage in a non-spontaneous event. Charge analysis shows there is a decrease in electron density of the carbon atom up to the transition state. After the transition state is reached, its electron density increases. For the leaving group and the nucleophile, there is a constant increase and decrease in electron density throughout the reaction, respectively. All models, except the iodide ones, involve an early transition state, with HS^−^ being the earliest (41.8%). Although different ions present in soil and water may help degrade chloroacetanilide herbicides, the use and development of environmentally friendly sulfur compounds seems to be the best strategy for these pesticides’ environmental remediations.

## Figures and Tables

**Table 1:** Activation free energy in kcal/mol of the S_N_2 reaction of chloroacetanilides in water.

Nucleophile	Acetochlor	Alachlor	Metolachlor	Propachlor
*Br^−^*	23.81	23.84	22.98	22.18
*I^−^*	21.80	22.04	22.53	21.22
*HS^−^*	20.45	19.33	20.25	18.36
*S_2_O_3_^−2^ (S)*	18.73	20.08	20.29	19.09
*S_2_O_3_^−2^ (O)*	26.28	26.37	26.77	26.68

**Table 2:** Average evolution percentage (%Evav) and synchronicity (Sy) for the nucleophilic substitution of alachlor and propachlor.

System		Br^−^	I^−^	HS^−^	S_2_O_3_^−2^ (S)	S_2_O_3_^−2^ (S)
*Alachlor*	*%Evav*	49.1	56.8	40.8	45.0	45.8
	*Sy*	0.904	0.928	0.899	0.892	0.839
*Propachlor*	*%Evav*	49.2	56.4	41.2	46.4	46.5
	*Sy*	0.903	0.917	0.895	0.892	0.833

## Supplementary Materials

The following are available online at https://www.mdpi.com/article/10.3390/ijms22136876/s1.
