# Supplementary material for: Effect of the Nucleophile’s Nature on Chloroacetanilide Herbicides Cleavage Reaction Mechanism. A DFT Study
Source: Int J Mol Sci. 2021 Jun 26;22(13):6876. doi: 10.3390/ijms22136876 (PMC8268095; doi:10.3390/ijms22136876)
Supplement: Supplementary file 1 [file ijms-22-06876-s001.zip › ijms-1264778-supplementary.pdf]

International Journal of *Molecular Sciences*

MDPI

Article

Effect of the Nucleophile´s Nature on Chloroacetanilide Herbicides Cleavage Reaction Mechanism. A DFT Study

Sebastián A. Cuesta [1,2], F. Javier Torres [1,2], Luis Rincón [1,2], José Luis Paz [3], Edgar A. Márquez [4,*] and José Ramón Mora [1,2,*]

1. Grupo de Química Computacional y Teórica (QCT-USFQ), Departamento de Ingeniería Química, Universidad San Francisco de Quito, Diego de Robles y Vía Interoceánica, Quito 170901, Ecuador; sebascuestahoyos89@gmail.com (S.A.C.); jtorres@usfq.edu.ec (F.J.T.); lrincon@usfq.edu.ec (L.R.)
2. Departamento de Ingeniería Química, Instituto de Simulación Computacional (ISC-USFQ), Universidad San Francisco de Quito, Diego de Robles y Vía Interoceánica, Quito 170901, Ecuador. sebascuestahoyos89@gmail.com (S.A.C.); jtorres@usfq.edu.ec (F.J.T.); lrincon@usfq.edu.ec (L.R.)
3. Departamento Académico de Química Inorgánica, Facultad de Química e Ingeniería Química, Universidad Nacional Mayor de San Marcos, Cercado de Lima 15081, Perú; jpazr@unmsm.edu.pe (J.L.P)
4. Grupo de Investigaciones en Química y Biología, Departamento de Química y Biología, Facultad de Ciencias Exactas, Universidad del Norte, Carrera 51B, Km 5, vía Puerto Colombia, Barranquilla 081007, Colombia.

* Correspondence: jrmora@usfq.edu.ec (J.R.M.); ebrazon@uninorte.edu.co (E.A.M.)

SUPPLEMENTARY MATERIAL

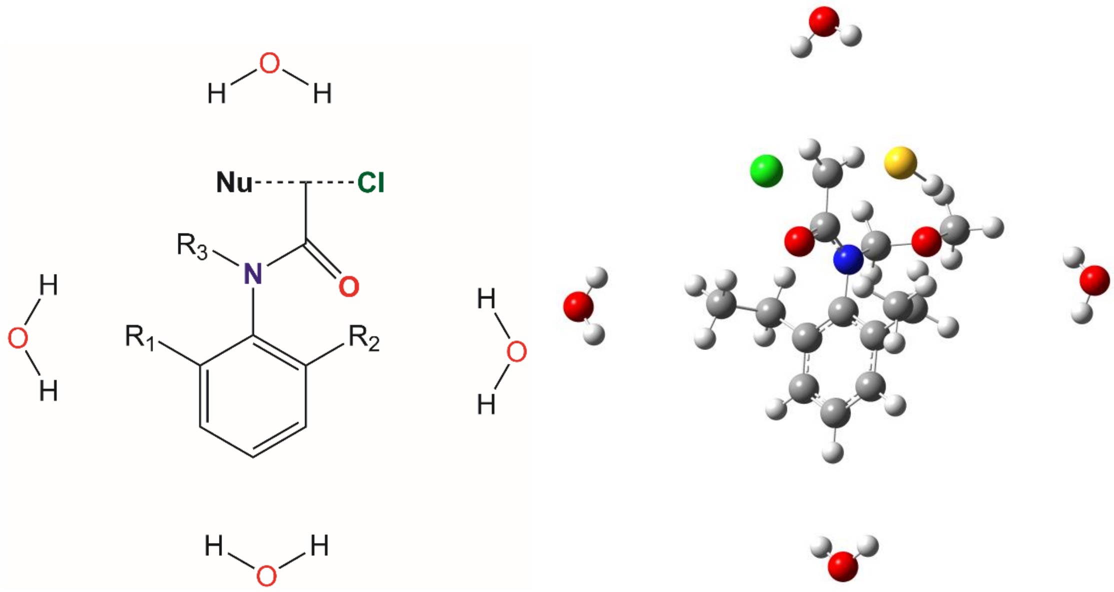

Figure S1. 2D and 3D representation of the 4-water model in the TS structure of Chloroacetanilide Herbicides

Table S1. Solvation electronic energy of chloroacetanilides and transition states

Chloroacetanilide	Nucleophile	Solvation Energy (kcal/mol)
**Alachlor**	*(Reagent)*	1.7
	Br^- *(TS)*	0.8
	I^- *(TS)*	1.3
	HS^- *(TS)*	1.2
	$S_2O_3^{-2}$ *(S) (TS)*	1.1
	$S_2O_3^{-2}$ *(O) (TS)*	1.0
**Acetochlor**	*(Reagent)*	1.3
	Br^- *(TS)*	0.7
	I^- *(TS)*	1.2
	HS^- *(TS)*	1.0
	$S_2O_3^{-2}$ *(S) (TS)*	1.2
	$S_2O_3^{-2}$ *(O) (TS)*	1.5
**Propachlor**	*(Reagent)*	1.5
	Br^- *(TS)*	0.4
	I^- *(TS)*	0.8
	HS^- *(TS)*	0.3
	$S_2O_3^{-2}$ *(S) (TS)*	1.2
	$S_2O_3^{-2}$ *(O) (TS)*	0.3
**Metolachlor**	*(Reagent)*	0.0
	Br^- *(TS)*	-0.3
	I^- *(TS)*	-0.4
	HS^- *(TS)*	0.7
	$S_2O_3^{-2}$ *(S) (TS)*	0.2
	$S_2O_3^{-2}$ *(O) (TS)*	0.9

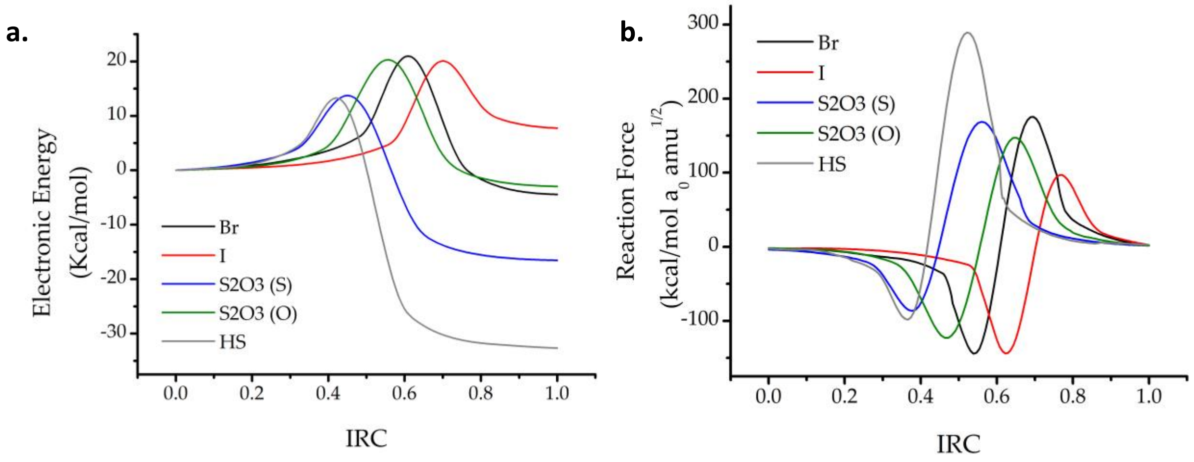

Figure S2. IRC (a) and RF (b) plots for the nucleophilic substitution of acetochlor

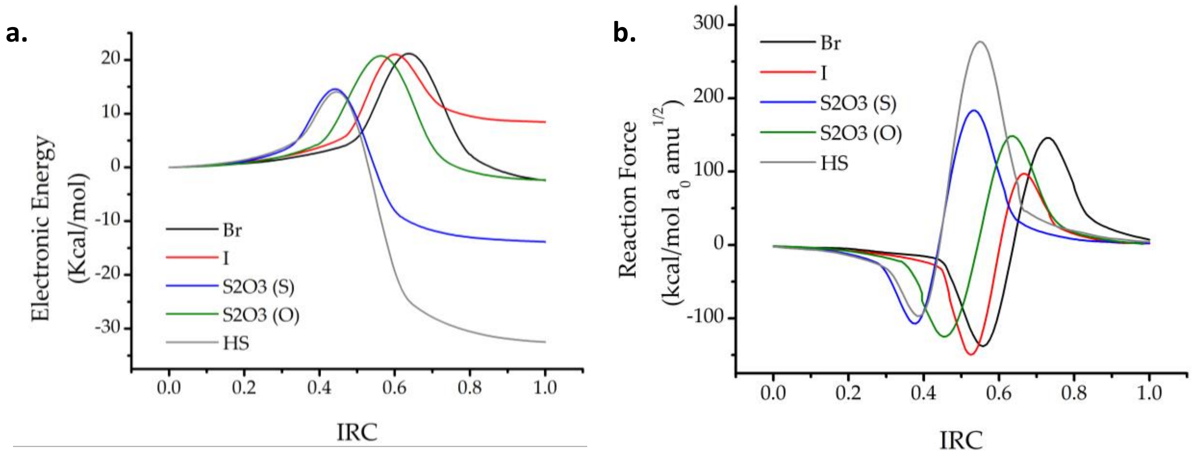

Figure S3. IRC (a) and RF (b) plots for the nucleophilic substitution of metolachlor.

Table S2. Reaction force Works (in Kcal/mol) involved in the nucleophilic substitution of propachlor

Nucleophile	*w1*	*w2*	*w3*	*w4*
Br^-	13.6	8.0	-11.0	-14.6
I^-	11.9	7.9	-4.4	-7.8
HS^-	9.1	4.4	-22.4	-26.2
$S_2O_3^{-2}$ *(S)*	9.4	5.1	-10.9	-18.0
$S_2O_3^{-2}$ *(O)*	12.5	8.7	-10.0	-14.3

Table S3. Reaction force Works (in Kcal/mol) involved in the nucleophilic substitution of metolachlor

Nucleophile	*w1*	*w2*	*w3*	*w4*
Br^-	13.1	8.0	-9.1	-14.4
I^-	13.0	8.0	-4.5	-8.1
HS^-	9.6	4.4	-19.2	-27.3
$S_2O_3^{-2}$ (S)	9.3	5.1	-12.4	-15.9
$S_2O_3^{-2}$ (O)	13.1	7.4	-10.2	-12.9

Table S4. Reaction force Works (in Kcal/mol) involved in the nucleophilic substitution of alachlor

Nucleophile	*w1*	*w2*	*w3*	*w4*
Br^-	12.3	7.7	-9.5	-15.8
I^-	11.8	8.1	-4.6	-7.8
HS^-	8.9	4.3	-20.4	-29.2
$S_2O_3^{-2}$ (S)	8.9	5.0	-12.9	-17.8
$S_2O_3^{-2}$ (O)	12.6	7.5	-10.3	-12.9

Table S5. Reaction force Works (in Kcal/mol) involved in the nucleophilic substitution of acetochlor

Nucleophile	*w1*	*w2*	*w3*	*w4*
Br^-	13.2	7.7	-11.1	-14.2
I^-	11.7	8.1	-4.6	-7.7
HS^-	8.7	4.5	-19.6	-26.1
$S_2O_3^{-2}$ (S)	8.7	5.0	-12.9	-17.3
$S_2O_3^{-2}$ (O)	12.6	7.5	-10.4	-12.8

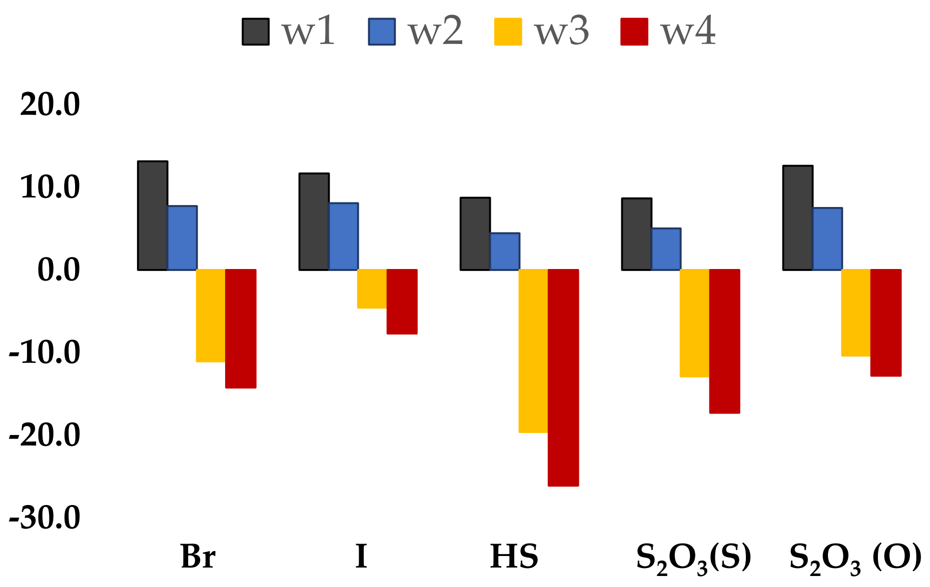

Figure S4. Reaction force work 1 and 2 for the nucleophilic substitution reaction of acetochlor.

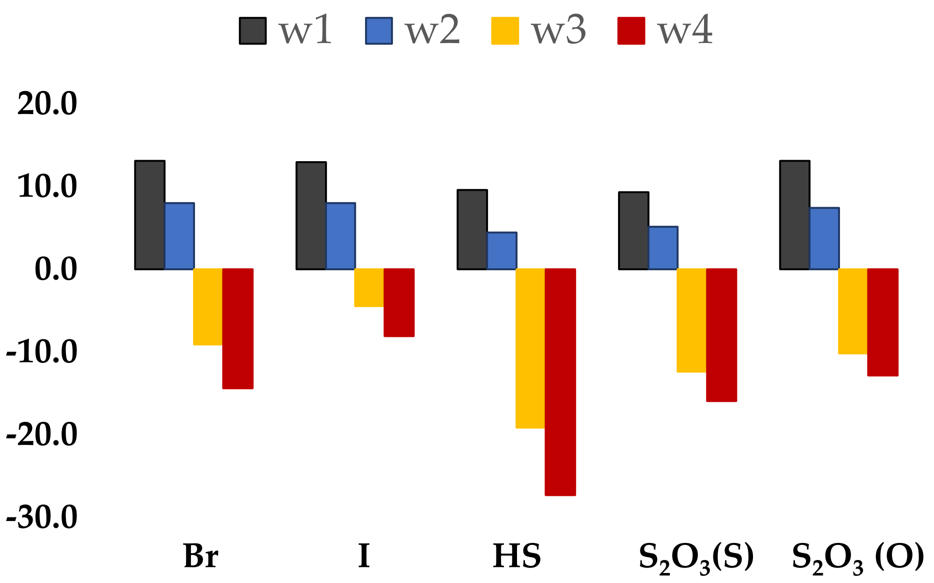

Figure S5. Reaction force work 1 and 2 for the nucleophilic substitution reaction of metolachlor.

a. b.

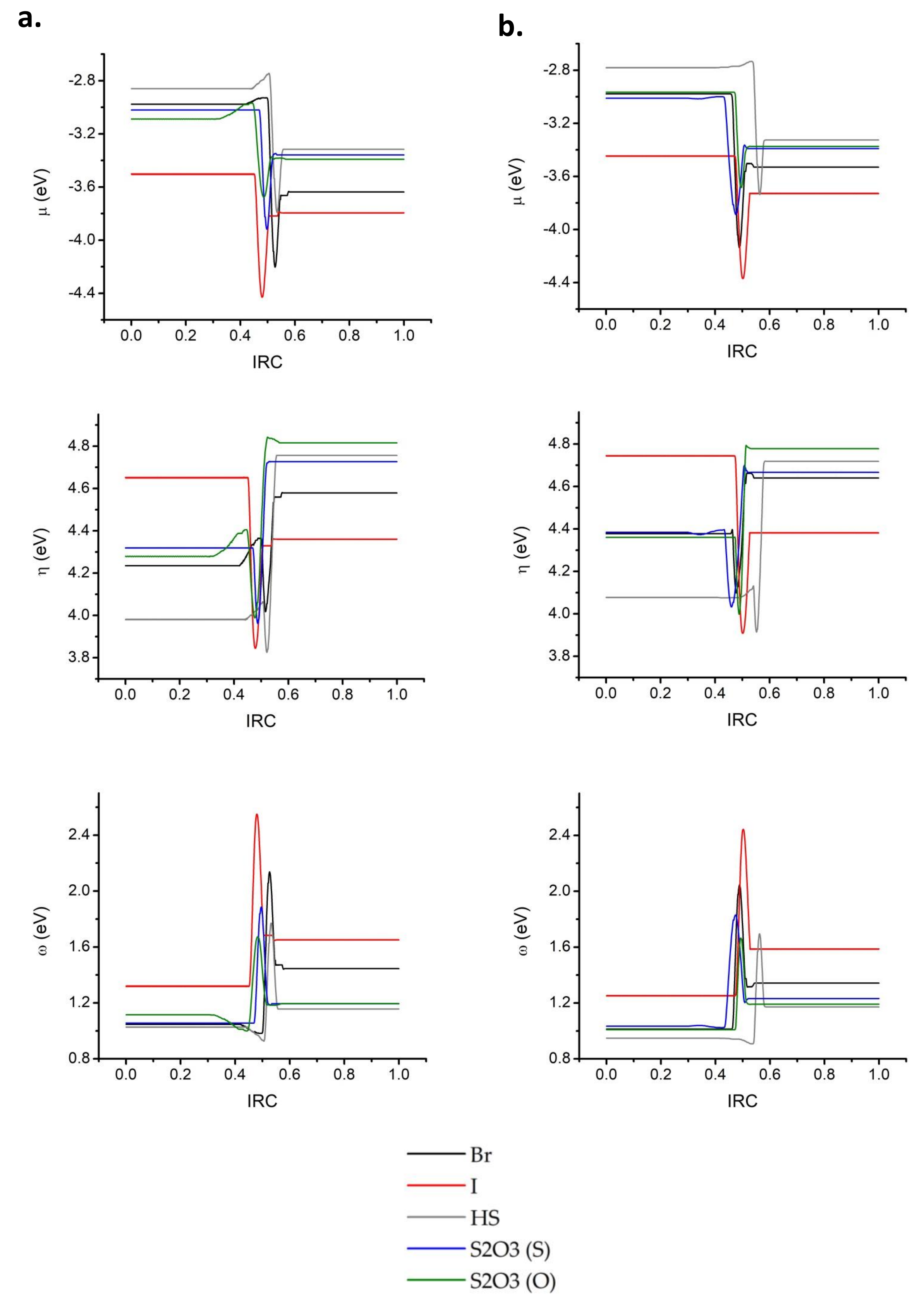

Figure S6. μ, η, and ω plots for the nucleophilic substitution of alachlor (a) and propachlor (b).

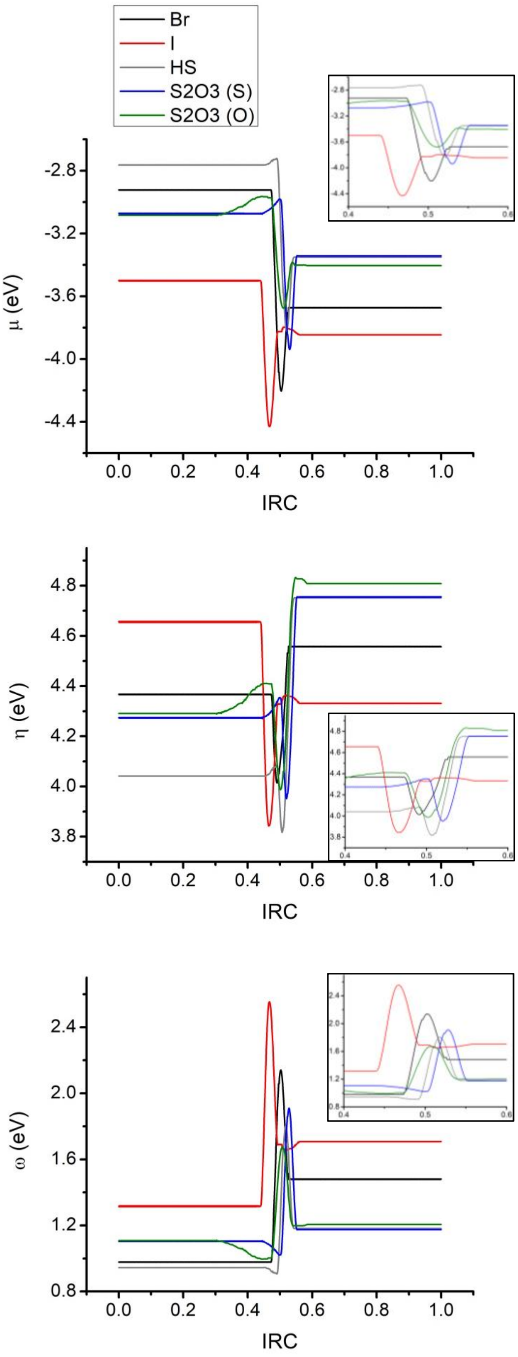

Figure S7. μ, η, and ω plots for the nucleophilic substitution of acetochlor.

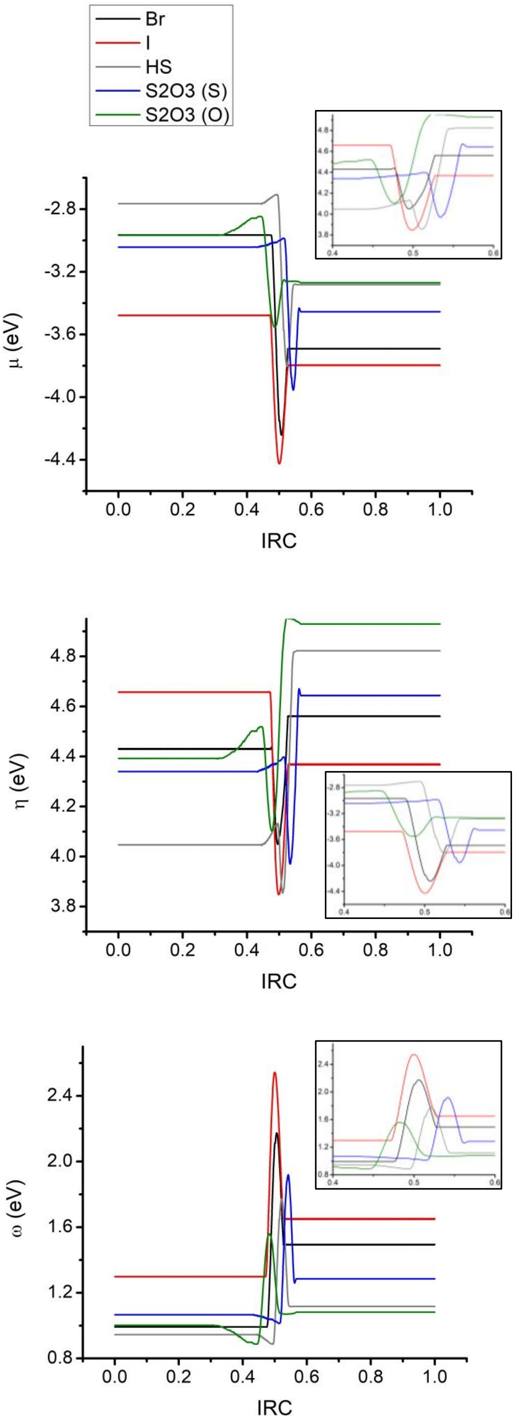

Figure S8. μ, η, and ω plots for the nucleophilic substitution of metolachlor.

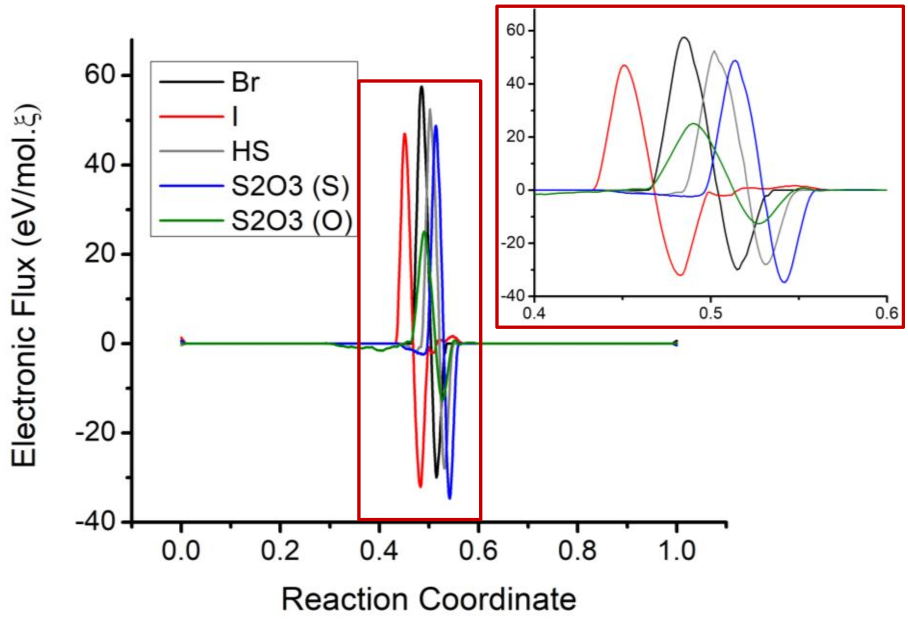

Figure S9. REF plots for the nucleophilic substitution of acetochlor.

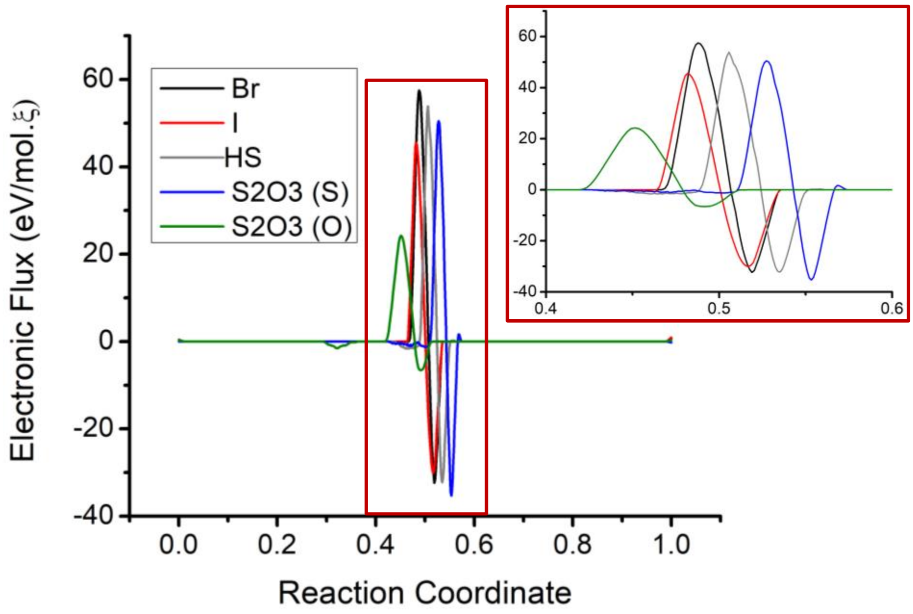

Figure S10. REF plots for the nucleophilic substitution of metolachlor.

Table S6. Charge analysis of the nucleophilic attack mechanism of Acetochlor

Nucleophile		Cl	C	Nu
Br^-	*R*	-0.123	-0.397	-1.000
	TS	-0.577	-0.330	-0.576
	P	-1.000	-0.489	-0.034
	δQi	0.454	-0.067	-0.424
I^-	*R*	-0.123	-0.397	-1.000
	TS	-0.622	-0.382	-0.500
	P	-1.000	-0.630	0.108
	δQi	0.499	-0.015	-0.500
HS^-	*R*	-0.123	-0.397	-1.108
	TS	-0.509	-0.356	-0.744
	P	-1.000	-0.557	-0.121
	δQi	0.386	-0.041	-0.364
$S_2O_3^{-2}$ (S)	*R*	-0.123	-0.397	-0.843
	TS	-0.541	-0.356	-0.545
	P	-1.000	-0.584	-0.089
	δQi	0.418	-0.041	-0.298
$S_2O_3^{-2}$ (O)	*R*	-0.123	-0.397	-1.125
	TS	-0.606	-0.183	-0.981
	P	-1.000	-0.131	-0.794
	δQi	0.483	-0.214	-0.144

Table S7. Charge analysis of the nucleophilic attack mechanism of Alachlor

Nucleophile		Cl	C	Nu
Br^-	*R*	-0.123	-0.396	-1.000
	TS	-0.578	-0.329	-0.576
	P	-1.000	-0.488	-0.033
	δQi	0.455	-0.067	-0.424
I^-	*R*	-0.123	-0.396	-1.000
	TS	-0.621	-0.381	-0.500
	P	-1.000	-0.629	0.108
	δQi	0.498	-0.015	-0.500
HS^-	*R*	-0.123	-0.396	-1.108

	TS	-0.497	-0.357	-0.746
	P	-1.000	-0.558	-0.120
	δQi	0.374	-0.039	-0.362
**$S_2O_3^{-2}$ (S)**	*R*	-0.123	-0.396	-0.843
	TS	-0.539	-0.354	-0.544
	P	-1.000	-0.584	-0.089
	δQi	0.416	-0.042	-0.299
**$S_2O_3^{-2}$ (O)**	*R*	-0.123	-0.396	-1.125
	TS	-0.607	-0.181	-0.981
	P	-1.000	-0.131	-0.794
	δQi	0.484	-0.215	-0.144

Table S8. Charge analysis of the nucleophilic attack mechanism of Metolachlor

Nucleophile		Cl	C	Nu
**Br^-**	*R*	-0.124	-0.391	-1.000
	TS	-0.586	-0.325	-0.569
	P	-1.000	-0.483	-0.035
	δQi	0.462	-0.066	-0.431
**I^-**	*R*	-0.124	-0.391	-1.000
	TS	-0.629	-0.375	-0.497
	P	-1.000	-0.624	0.107
	δQi	0.505	-0.016	-0.503
**HS^-**	*R*	-0.124	-0.391	-1.108
	TS	-0.511	-0.349	-0.737
	P	-1.000	-0.552	-0.122
	δQi	0.387	-0.042	-0.371
**$S_2O_3^{-2}$ (S)**	*R*	-0.124	-0.391	-0.843
	TS	-0.555	-0.349	-0.551
	P	-1.000	-0.579	-0.088
	δQi	0.431	-0.042	-0.292
**$S_2O_3^{-2}$ (O)**	*R*	-0.124	-0.391	-1.125
	TS	-0.616	-0.179	-0.989
	P	-1.000	-0.129	-0.794
	δQi	0.492	-0.212	-0.136

Table S9. Charge analysis of the nucleophilic attack mechanism of Propachlor

Nucleophile		Cl	C	Nu
**Br^-**	*R*	-0.128	-0.394	-1.000
	TS	-0.586	-0.321	-0.581
	P	-1.000	-0.486	-0.040
	δQi	0.458	-0.073	-0.419
**I^-**	*R*	-0.128	-0.394	-1.000
	TS	-0.631	-0.372	-0.503
	P	-1.000	-0.633	0.098
	δQi	0.503	-0.022	-0.497
**HS^-**	*R*	-0.128	-0.394	-1.108
	TS	-0.509	-0.345	-0.740
	P	-1.000	-0.556	-0.123
	δQi	0.381	-0.049	-0.368
**$S_2O_3^{-2}$ (S)**	*R*	-0.128	-0.394	-0.843
	TS	-0.561	-0.345	-0.548
	P	-1.000	-0.583	-0.090
	δQi	0.433	-0.049	-0.295
**$S_2O_3^{-2}$ (O)**	*R*	-0.128	-0.394	-1.125
	TS	-0.623	-0.174	-0.987
	P	-1.000	-0.130	-0.793
	δQi	0.495	-0.220	-0.138

Table S10. Wiberg bond indexes (Bi), evolution percent, synchronicity (Sy) and average value (%Evav) for the nucleophilic substitution of acetochlor

		Wiberg bond index (Bi)		Sy	%Evav
		B(C-Cl)	*B(C-Nu)*		
**Br^-**	*R*	0.979	0.000	0.904	48.9
	TS	0.453	0.437		
	P	0.000	0.987		
	%Evi	53.67	44.23		
		B(C-Cl)	*B(C-Nu)*		
**I^-**	*R*	0.979	0.000	0.928	56.7
	TS	0.384	0.513		
	P	0.000	0.974		
	%Evi	60.80	52.61		

		$B_{(C-Cl)}$	$B_{(C-Nu)}$		
**HS^-**	*R*	0.979	0.000		
	TS	0.528	0.376	0.897	41.8
	P	0.000	1.003		
	%Evi	46.09	37.46		
		$B_{(C-Cl)}$	$B_{(C-Nu)}$		
**$S_2O_3^{-2}$ (S)**	*R*	0.979	0.000		
	TS	0.489	0.398	0.892	45.2
	P	0.000	0.987		
	%Evi	50.08	40.35		
		$B_{(C-Cl)}$	$B_{(C-Nu)}$		
	R	0.979	0.000		
**$S_2O_3^{-2}$ (O)**	*TS*	0.460	0.331	0.839	45.7
	P	0.000	0.863		
	%Evi	53.03	38.32		

Table S11. Wiberg bond indexes (Bi), evolution percent, synchronicity (Sy) and average value (%Evav) for the nucleophilic substitution of alachlor

		**Wiberg bond index (Bi)**		**Sy**	**%Evav**
		$B_{(C-Cl)}$	$B_{(C-Nu)}$		
**Br^-**	*R*	0.978	0.000		
	TS	0.452	0.438	0.904	49.1
	P	0.000	0.987		
	%Evi	53.80	44.41		
		$B_{(C-Cl)}$	$B_{(C-Nu)}$		
**I^-**	*R*	0.978	0.000		
	TS	0.383	0.513	0.928	56.8
	P	0.000	0.974		
	%Evi	60.82	52.69		
		$B_{(C-Cl)}$	$B_{(C-Nu)}$		
**HS^-**	*R*	0.978	0.000		
	TS	0.539	0.368	0.899	40.8
	P	0.000	1.002		
	%Evi	44.93	36.69		
**$S_2O_3^{-2}$ (S)**		$B_{(C-Cl)}$	$B_{(C-Nu)}$		
	R	0.978	0.000	0.892	45.0
	TS	0.490	0.397		

	P	0.000	0.987		
	%Evi	49.92	40.18		
		$B_{(C-Cl)}$	$B_{(C-Nu)}$		
	R	0.978	0.000		
**$S_2O_3^{-2}$ (O)**	*TS*	0.459	0.331	0.839	45.8
	P	0.000	0.862		
	%Evi	53.11	38.40		

Table S12. Wiberg bond indexes (Bi), evolution percent, synchronicity (Sy) and average value (%Evav) for the nucleophilic substitution of metolachlor

		**Wiberg bond index (Bi)**		**Sy**	**%Evav**
		$B_{(C-Cl)}$	$B_{(C-Nu)}$		
**Br^-**	*R*	0.976	0.000		
	TS	0.441	0.446	0.904	50.0
	P	0.000	0.985		
	%Evi	54.84	45.21		
		$B_{(C-Cl)}$	$B_{(C-Nu)}$		
**I^-**	*R*	0.976	0.000		
	TS	0.377	0.517	0.928	57.3
	P	0.000	0.973		
	%Evi	61.40	53.14		
		$B_{(C-Cl)}$	$B_{(C-Nu)}$		
**HS^-**	*R*	0.976	0.000		
	TS	0.525	0.377	0.898	42.0
	P	0.000	1.001		
	%Evi	46.26	37.67		
		$B_{(C-Cl)}$	$B_{(C-Nu)}$		
	R	0.976	0.000		
**$S_2O_3^{-2}$ (S)**	*TS*	0.474	0.410	0.893	46.5
	P	0.000	0.986		
	%Evi	51.48	41.52		
		$B_{(C-Cl)}$	$B_{(C-Nu)}$		
	R	0.976	0.000		
**$S_2O_3^{-2}$ (O)**	*TS*	0.446	0.333	0.832	46.5
	P	0.000	0.860		
	%Evi	54.28	38.65		

Table S13. Wiberg bond indexes (Bi), evolution percent, synchronicity (Sy) and average value (%Evav) for the nucleophilic substitution of propachlor

		Wiberg bond index (Bi)		Sy	%Evav
		$B_{(C\text{-}Cl)}$	$B_{(C\text{-}Nu)}$		
**Br^-**	*R*	0.978	0.000		
	TS	0.450	0.438	0.903	49.2
	P	0.000	0.987		
	%Evi	53.94	44.38		
		$B_{(C\text{-}Cl)}$	$B_{(C\text{-}Nu)}$		
**I^-**	*R*	0.978	0.000		
	TS	0.381	0.512	0.917	56.4
	P	0.000	0.991		
	%Evi	61.03	51.70		
		$B_{(C\text{-}Cl)}$	$B_{(C\text{-}Nu)}$		
**HS^-**	*R*	0.978	0.000		
	TS	0.532	0.370	0.895	41.2
	P	0.000	1.004		
	%Evi	45.56	36.87		
		$B_{(C\text{-}Cl)}$	$B_{(C\text{-}Nu)}$		
**$S_2O_3^{-2}$ (S)**	*R*	0.978	0.000		
	TS	0.475	0.409	0.892	46.4
	P	0.000	0.989		
	%Evi	51.39	41.35		
		$B_{(C\text{-}Cl)}$	$B_{(C\text{-}Nu)}$		
	R	0.978	0.000		
**$S_2O_3^{-2}$ (O)**	*TS*	0.447	0.334	0.833	46.5
	P	0.000	0.862		
	%Evi	54.24	38.73		
